# Microspore culture reveals complex meiotic behaviour in a trigenomic *Brassica* hybrid

**DOI:** 10.1186/s12870-015-0555-9

**Published:** 2015-07-08

**Authors:** Annaliese S. Mason, Junko Takahira, Chhaya Atri, Birgit Samans, Alice Hayward, Wallace A. Cowling, Jacqueline Batley, Matthew N. Nelson

**Affiliations:** School of Agriculture and Food Sciences, The University of Queensland, Brisbane, 4072 Australia; Centre for Integrative Legume Research, The University of Queensland, Brisbane, 4072 Australia; Department of Plant Breeding, IFZ Research Centre for Biosystems, Land Use and Nutrition, Justus Liebig University, Heinrich-Buff-Ring 26-32, 35392 Giessen, Germany; School of Plant Biology, The University of Western Australia, 35 Stirling Highway, Crawley, 6009 Perth, Australia; Plant Breeding & Genetics Department, Punjab Agricultural University, Ferozepur Road, Ludhiana, Punjab 141004 India; The UWA Institute of Agriculture, The University of Western Australia, 35 Stirling Highway, Crawley, 6009 Perth, Australia

**Keywords:** Allohexaploid, *Brassica*, SNP chip, Chromosome behaviour, Allele copy number analysis, Interspecific hybrids

## Abstract

**Background:**

Development of synthetic allohexaploid *Brassica* (2n = AABBCC) would be beneficial for agriculture, as allelic contributions from three genomes could increase hybrid vigour and broaden adaptation. Microspore culture of a near-allohexaploid hybrid derived from the cross (*B. napus* × *B. carinata*) × *B. juncea* was undertaken in order to assess the frequency and distribution of homologous and homoeologous crossovers in this trigenomic hybrid. SNP and SSR molecular markers were used to detect inheritance of A, B and C genome alleles in microspore-derived (MD) progeny. SNP allele copy number was also assessed. The MD progeny were also compared to progeny derived by self-pollination and open-pollination for fertility (estimated by self-pollinated seed set and pollen viability) and DNA ploidy (measured by flow cytometry).

**Results:**

In the trigenomic hybrid, homologous chromosome pairs A^j^-A^n^, B^j^-B^c^ and C^n^-C^c^ had similar meiotic crossover frequencies and segregation to that previously observed in established *Brassica* species, as demonstrated by marker haplotype analysis of the MD population. Homoeologous pairing between chromosomes A1-C1, A2-C2 and A7-C6 was detected at frequencies of 12–18 %, with other homoeologous chromosome regions associating from 8 % (A3-C3) to 0–1 % (A8-C8, A8-C9) of the time. Copy number analysis revealed eight instances of additional chromosomes and 20 instances of chromosomes present in one copy in somatically doubled MD progeny. Presence of chromosome A6 was positively correlated with self-pollinated seed set and pollen viability in the MD population. Many MD progeny were unable to produce self-pollinated seed (76 %) or viable pollen (53 %), although one MD plant produced 198 self-pollinated seeds. Average fertility was significantly lower in progeny obtained by microspore culture than progeny obtained by self-pollination or open-pollination, after excluding MD progeny which had not undergone chromosome doubling.

**Conclusions:**

Based on SNP data analysis of the microspore-derived progeny, crossover frequency per chromosome in the allohexaploid hybrid was found to be similar to that in established *Brassica* species, suggesting that the higher chromosome number did not significantly disrupt cellular regulation of meiosis. SNP allele copy number analysis revealed the occurrence not only of homoeologous duplication/deletion events but also other cryptic duplications and deletions that may have been the result of mitotic instability. Microspore culture simplified the assessment of chromosome behaviour in the allohexaploid hybrid but yielded progeny with lower fertility and a greater range of ploidy levels compared to progeny obtained by self- or open-pollination.

**Electronic supplementary material:**

The online version of this article (doi:10.1186/s12870-015-0555-9) contains supplementary material, which is available to authorized users.

## Background

The *Brassica* genus comprises a large number of cultivated crop species, including oilseeds (canola, rapeseed), vegetables (cabbage, turnip, broccoli, cauliflower, pak choi, buk choy) and condiments (mustards) [1]. Six of the cultivated *Brassica* species share a unique genomic relationship. *Brassica rapa* (2n = 2x = AA = 20), *B. nigra* (2n = 2x = BB = 16) and *B. oleracea* (2n = 2x = 18) are diploids. Ancestral hybridisation events between these species gave rise to the allotetraploid species *B. juncea* (2n = 4x = AABB = 36), *B. napus* (2n = 4x = AACC = 38) and *B. carinata* (2n = 4x = BBCC = 34) [2, 3]. Due to this genomic relationship, there exists great potential for transfer of useful alleles between these species for agricultural benefit [4]. The allotetraploid crops (canola, rapeseed, mustards) can be recreated from the modern-day diploids to broaden their genetic bases for breeding purposes [5]. This involves introgressing novel genetic variation and useful alleles from the diploid species, which include many wild species, countering recent reductions in genetic diversity in the crop-type allopolyploids [5–7]. In addition, a new species could potentially be created with genomic composition 2n = 6x = AABBCC = 54 from crosses between the diploids and/or allotetraploids, with greater potential for allelic heterosis and hence hybrid vigour [4].

However, a major obstacle to the success of these breeding approaches is the high rate of abnormal meiosis in resynthesised *Brassica* allopolyploids [5, 8]. This has been demonstrated in both synthetic *B. napus* (2n = AACC) recreated from crosses between *B. rapa* (2n = AA) and *B. oleracea* (2n = CC) [8, 9], as well as in allohexaploids (2n = AABBCC) created from crosses between *B. rapa* (2n = AA) and *B. carinata* (2n = BBCC) [10, 11]. This meiotic instability manifests as non-homologous interactions between the closely related A- and C-genome chromosomes during meiosis [12–14], and results in loss of chromosomes, instability of generational inheritance and infertility in subsequent generations [15–17]. In contrast, *B. napus* (2n = AACC) is a functionally diploid species, with a regular disomic mode of chromosome inheritance [18, 19], despite the close relationship of the A and C genomes [20]. This improved genetic control may have arisen soon after the formation of *B. napus* via mutation resulting in novel genetic variation or by accumulation of minor alleles inherited from the parent diploids. Alternatively, the diploid progenitors of the first *B. napus* lines may have had inherently greater genetic control of meiosis than the *B. rapa* and/or *B. oleracea* germplasm used to experimentally resynthesise *B. napus*. Quantitative trait loci (QTL) contributing to genetic regulation of meiotic behaviour in *B. napus* have been identified [21, 22]. However, strong qualitative effects on homoeologous pairing, such as those observed for the *Ph1* locus of wheat [23], have yet to be discovered.

Previously, we developed a novel method for generating allohexaploid *Brassica* using only allotetraploid species [24]. In a two-step process, crosses between *B. juncea*, *B. napus* and *B. carinata* generated a novel near-allohexaploid plant with the hypothesis that they would inherit meiotic stability alleles from each of the natural allotetraploid species [24]. Hypothetically, meiotic stability alleles from natural allotetraploid *Brassica* species may be effective in regulating homologous chromosome pairing and transmission in this synthetic allohexaploid *Brassica* hybrid. We also assessed A- and C-genome allele and chromosome transmission in an F_2_ population derived from this F_1_ hybrid [25].

**Fig. 1:**
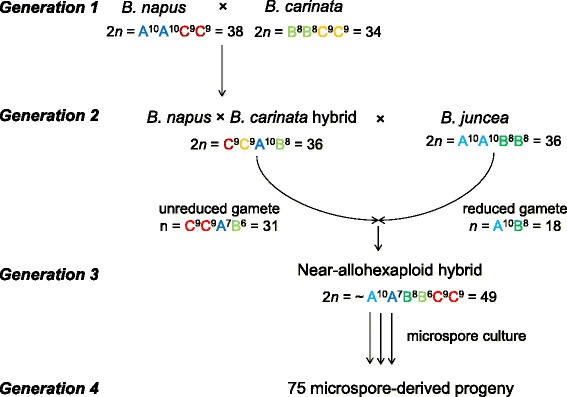
The four-generation (Generation 1 to Generation 4) pedigree used in this experiment. Interspecific hybridisation between *B. napus* and *B. carinata* (Generation 1) generated an unbalanced CCAB hybrid (Generation 2). An unreduced, aneuploid gamete from the CCAB hybrid combined with a reduced gamete from *B. juncea* to produce a near-allohexaploid hybrid (Generation 3) missing three *B. napus* A-genome chromosomes and two *B. carinata* B-genome chromosomes. The near-allohexaploid was subjected to microspore culture to produce a population of 75 microspore-derived progeny (Generation 4)

In this study, we developed 75 microspore-derived (MD) progeny from the novel near-allohexaploid hybrid (Fig. 1) and genotyped these progeny using the Illumina 60K Brassica SNP chip and SSR markers. Hence, homologous and homoeologous crossover frequency and distribution across and between each of the A, B and C genomes could be assessed for the first time in a trigenomic *Brassica* hybrid. DNA content (flow cytometry and allele copy number) and fertility (self-seed set and pollen viability) data were also collected and compared to 50 self-pollinated ([25], previously published data) and 50 open-pollinated (OP) progeny derived from the same hybrid.

## Results

### Genome coverage of molecular markers

Of the 52157 SNP markers on the *Brassica* 60K Infinium SNP chip, 7651 were selected on the basis of polymorphism between the parental lines and lack of evidence of cross-amplification between the A and C genomes in this population. The set of SNP markers used in this study was manually clustered in Genome Studio for these genotypes previously (see [25]) and markers that showed more than three genotype clusters or were heterozygous in the control genotypes (indicative of cross-amplification between the A and C genomes) were removed. In total, 2667 markers in the A genome and 4984 in the C genome were retained; with polymorphic SNP markers per chromosome ranging from 123 to 1283 (average 402 per chromosome) (Additional file 1: Table S1). SNP genotyping of the population of 75 microspore-derived (MD) progeny showed that 74 MD progeny had only one parental allele at most loci, indicating that these progeny were derived from reduced (haploid) gametes. One MD progeny (MD_047, Additional file 1: Table S1) was heterozygous at most A- and C-genome loci, consistent with its derivation from an unreduced gamete by first division restitution; this individual was removed from subsequent analyses. Three pairs of individuals had identical molecular marker results, consonant with their production from twinned embryos during the microspore culture process [26]. One individual from each pair was removed from subsequent analyses (MD_012, MD_056 and MD_070).

Of the 163 SSR primer pairs screened, 55 detected polymorphisms in the B genome between the *B. juncea* and *B. carinata* parent genotypes, amplifying 90 B-genome specific alleles. Of these 90 B-genome marker alleles, 49 were contributed by the *B. juncea* parent and 41 by the *B. carinata* parent. Coverage of the *B. juncea* and *B. carinata* B-genome chromosomes ranged from 4 to 8 SSR loci per chromosome (Additional file 2: Table S2).

### Molecular karyotype of the near-allohexaploid hybrid

**Fig. 2:**
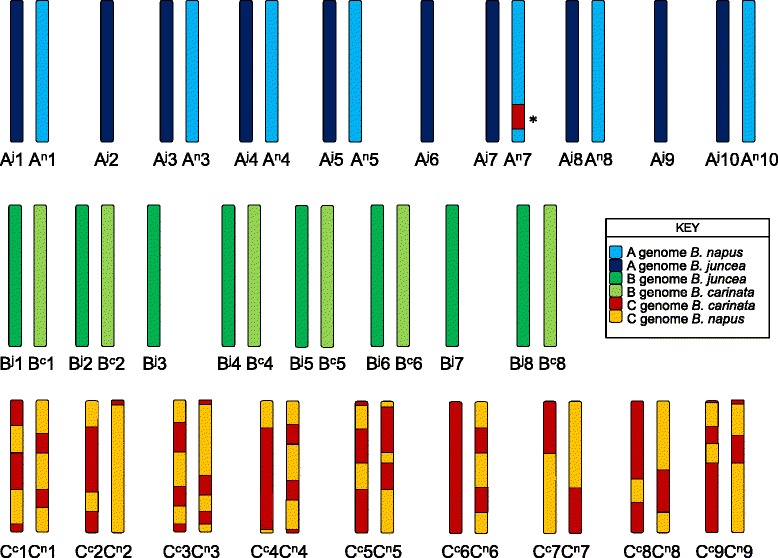
Graphical representation of the chromosome composition of the near-allohexaploid hybrid used in this experiment, inferred from molecular karyotyping of a four-generation pedigree (Fig. 1). The asterisk indicates a homoeologous non-reciprocal translocation from C^c^6 to A^n^7. Note that homologous recombination occurred in the CCAB hybrid (Generation 2) to produce recombinant C-genome chromosomes

The molecular karyotype of the near-allohexaploid hybrid as deduced from the presence and absence of parental SNP and SSR marker alleles in the four-generation pedigree is shown in Fig. 2. Inheritance of marker alleles in the microspore-derived population was used to infer the molecular karyotype of the near-allohexaploid hybrid.

All *B. juncea* A-genome (A^j^) chromosomes were present in the near-allohexaploid hybrid, but three *B. napus* A-genome chromosomes (A^n^2, A^n^6 and A^n^9) were lost in the formation of the AABBCC hybrid (between Generations 2 and 3; Figs. 1 and 2, Additional file 1: Table S1). All other A-genome chromosomes were present and unrecombined, except for an A^n^7 – C^c^6 homoeologous non-reciprocal translocation (Fig. 2), a duplication of part of chromosome A^n^10 that appeared to be pre-existing in the parental *B. napus* line, as described previously [25]; and a small C^c^4 duplication – A^n^4 deletion. All eight *B. juncea* B-genome (B^j^) chromosomes were present in the near-allohexaploid hybrid (Fig. 1; Additional file 2: Table S2), but two *B. carinata* chromosomes (B^c^3 and B^c^7) were lost, with no alleles present from either chromosome (Fig. 2). All C-genome chromosomes were transmitted to the near-allohexaploid hybrid (Fig. 2). Extensive homologous recombination between the *B. napus* and *B. carinata* C genomes (C^n^ and C^c^ respectively) in the C^n^C^c^A^n^B^c^ hybrid resulted in fixation (homozygosity) of 22 % of C genome loci in the MD population (Fig. 2; Additional file 1: Table S1). Recombinant C^n^/C^c^ chromosomes resulting from normal homologous recombination in the C^n^C^c^A^n^B^c^ hybrid meiosis were also detected in the MD population as sudden reversals of allelic scoring phase (see notes in Additional file 1: Table S1, Fig. 2).

### Transmission of alleles to the MD population

The normality of meiosis in the near-allohexaploid hybrid was first assessed by measuring the transmission frequencies of marker alleles to the MD population (Additional file 3: Table S3). Transmission frequencies did not significantly deviate from the expected 50 % for 618 of the total 707 marker allele bins (87.5 %) distributed across the A genome (Additional file 4: Figure S1). The most significant deviations were observed for alleles located on the ends of chromosomes A7 and A10, where *B. napus* alleles were present more often than predicted by chance. Alleles located on the three A-genome chromosomes that were unpaired (A^j^2, A^j^6, A^j^9; Fig. 2) each behaved differently: A^j^9 alleles were retained more often than predicted by chance in the population (~70 % of the time), A^j^6 alleles were present as often as expected (~45 % of the time) while A^j^2 alleles were lost a little more often than expected (~present 35 % of the time) (Additional file 4: Figure S1). Excluding univalent chromosomes on which non-homologous recombination events had occurred, only inheritance of A^j^9 significantly deviated from expected inheritance ratios for a univalent chromosome in the A genome (Additional file 1: Table S1; Additional file 4: Figure S1).

Marker alleles located on pairs of B-genome chromosomes showed no detectable bias towards retention of either *B. juncea* or *B. carinata* alleles (Additional file 2: Table S2; Additional file 4: Figure S2). By contrast, univalent chromosomes B^j^3 and B^j^7 both showed highly significant deviation from expected transmission ratios: B^j^3 was preferentially retained (~95 % of the time) and B^j^7 was preferentially lost (present 25 % of the time) (Additional file 4: Figure S2).

Most *B. napus* and *B. carinata* C-genome marker alleles were transmitted normally from the near-allohexaploid hybrid to the MD population (481 out of 608 alleles (79.1 %); Additional file 3: Table S3; Additional file 4: Figure S3). Strong segregation distortion towards retention of *B. napus* alleles was observed at the bottom of chromosome C4 (Additional file 4: Figure S3). Other regions of segregation distortion were observed on chromosomes C1 and C7, with a more significant bias towards retention of *B. carinata* C-genome alleles at the top of chromosome C5 (Additional file 4: Figure S3).

### Copy number analysis of A, B and C genome chromosomes in the MD population

**Fig. 3:**
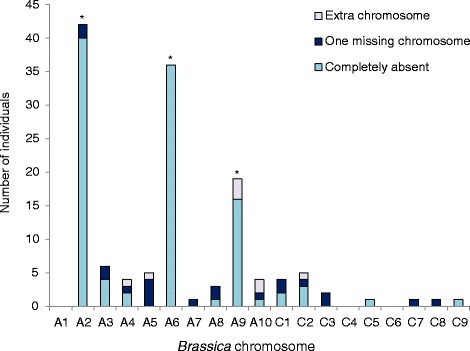
Relative chromosome copy number variation within each individual in a microspore-derived population resulting from a trigenomic *Brassica* hybrid. Asterisks indicate chromosomes with only one copy in the parent hybrid. One missing chromosome was only assessed in somatically-doubled “2n” progeny, and extra chromosome refers to either two copies in an “n” progeny, or three or four copies in a “2n” progeny

Standard SNP marker genotyping does not capture the full extent of homoeologous chromosome interactions, which can result in changes in allele copy numbers. Therefore, we conducted SNP marker allele copy number analysis to detect the deviations from regular chromosome transmission in the MD progeny. Deviations from expected copy numbers were indeed observed, including 8 instances of anomalous extra chromosomes and 20 instances of chromosomes present in only a single copy in “2n” progeny (Fig. 3). Duplication/deletion events involving partial chromosomes were also assessed. Twice as many deletion events (36) were observed in the population compared to duplication events (18), excluding the pre-existing duplication on chromosome A10 (25).

Unbalanced translocations, where a duplication of a homoeologous region was not accompanied by a corresponding deletion in another homoeologous region, were more common than balanced translocations (38, excluding chromosomes A7/C6 and A10). However, balanced translocations were far more likely to be observed than expected (p < 0.0001, Pearson’s *χ*^2^ test) assuming Mendelian segregation of a single homoeologous translocation event between two chromatids in a homoeologous set (e.g., A1/A1/C1/C1).

### Pairwise allele associations reveal regular homologous chromosome interactions

**Fig. 4:**
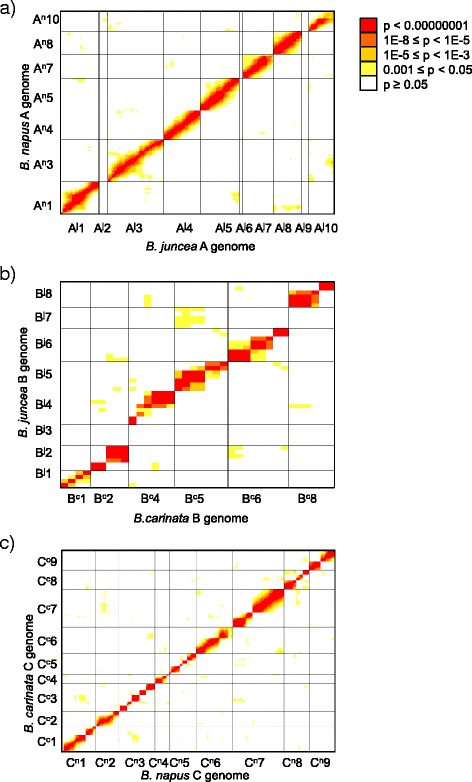
Allele segregation indicative of pairing between homologous (**a**) A-genome chromosomes (A^j^–A^n^), (**b**) B-genome chromosomes (B^j^–B^c^) and (**c**) C-genome chromosomes (C^n^–C^c^) in a near-allohexaploid hybrid. Allele segregation was assessed in a population of 71 progeny derived from microspores of a 2n = A^j^A^n^B^j^B^c^C^n^C^c^ hybrid. Only non-redundant SNP alleles are presented, arranged sequentially according to their genetic location (not drawn to scale). The Bonferroni multiple testing *p*-value correction for *p* < 0.05 significance is *p* < 0.000000050 in this analysis

The regularity of meiotic interactions between homologous chromosomes in the near-allohexaploid hybrid was assessed by detecting associations between all pairs of alleles segregating in the MD population, following the novel method described in [27]. All A^j^/A^n^ alleles for which a homologous chromosome pair was present (i.e., excluding A^j^2, A^j^6 and A^j^9; Fig. 2) segregated with high fidelity in the MD population, as evidenced by the strong diagonal in the allele association plot shown in Fig. 4a. Exceptions were the bottom third of chromosome A10 and an interstitial part of chromosome A7 (Fig. 4a). In the B genome, all B^j^ and B^c^ alleles for which a homologous chromosome pair was present (i.e., excluding B^j^3 and B^j^7) segregated with high fidelity (Fig. 4b). All C^n^/C^c^ alleles showed regular segregation and putative homologous pairing as assessed by allele transmission to the MD population (Fig. 4c), except for the bottom ends of C6 and C4.

The potential occurrence of meiotic interactions between non-homologous chromosomes was investigated using the same allele association approach. There was no statistical support for autosyndetic allele segregation (i.e., between non-homologous chromosomes from the same genome) as seen by the absence of off-diagonal associations in Fig. 4. Two significant allosyndetic allele segregation associations were observed: one association between parts of A7 and C6; and one terminal association (approximately 2 Mbp) between A4 and C4.

### Haplotype block analysis in the MD population reveals frequency of homologous and homoeologous meiotic recombination in the near-allohexaploid hybrid

Putative crossover events during meiosis in the near-allohexaploid hybrid were inferred by inspection of allele segregation patterns for each chromatid in the six parental subgenomes (A^j^, A^n^, B^c^, B^j^, C^c^ and C^n^) in the MD population. Recombination breakpoints were evidenced by the presence of alleles from one parental subgenome for only part of one chromatid; lack of recombination was evidenced either by complete presence or complete absence of all alleles from that parental subgenome on that chromatid for any given MD individual. If the presence of one allele perfectly matched the absence of another allele from a homologous or homoeologous genome, a recombination event was predicted to have occurred.

Univalent chromosomes A^j^2, A^j^6, A^j^9, B^j^3 and B^j^7 were transmitted intact 85 to 97 % of the time (Tables 1 and 2), but evidence for occasional homoeologous recombination events was found for every univalent chromosome (most commonly for A^j^2 and least commonly for B^j^3). Homologous chromosomes in the A, B and C genomes with no pre-existing duplications or rearrangements (i.e., excluding A7/C6 and A10) showed allele segregation indicative of normal homologous recombination with their homologue 83 to 100 % of the time (Tables 1 and 2). The highest degree of homoeologous recombination (12 to 18 %) was detected between chromosomes A1 and C1, between A2 and C2 and between A7 and C6 (Table 1).

**Table 1:** Summary of homologous and homoeologous chromosome pairing in the *Brassica* A and C genomes of a near allo-hexaploid hybrid from assessment of recombination breakpoints in 71 microspore-derived progeny

	A1	A2 ^a^	A3	A4	A5	A6 ^a^	A7	A8	A9 ^a^	A10
homologous	85 %	86 %	83 %	94 %	92 %	96 %	79 % (59 % ^b^)	99 %	89 %	96 % (76 % ^b^)
homoeologous	13 % (C1)	14 % (C2)	8 % (C3)	0 %	4 % (C5)	3 % (C5); 1 % (C7)	17 % (C6)	0 %	1 % (C8); 6 % (C9)	0 %
other^c^	3 %	0 %	8 %	5 %	4 %	0 %	5 %	1 %	4 %	4 %
	C1	C2	C3	C4	C5	C6	C7	C8	C9	
homologous	83 %	82 %	90 %	96 % (73 % ^b^)	90 %	68 %	97 %	99 %	90 %	
homoeologous	13 % (A1)	14 % (A2)	8 % (A3)	0 %	4 % (A5) 3 % (A6)	17 % (A7)	1 % (A6)	1 % (A9)	6 % (A9)	
other^c^	4 %	4 %	1 %	4 %	1 %	15 %	1 %	0 %	4 %	

^a^Univalent inheritance assessed rather than homologous pairing

^b^Including translocation region for which two copies of one parental allele were present, confounding assessment

^c^Recombination or non-segregation events which could not be unambiguously attributed to homoeologous recombination; includes probable univalent formation for homologous pairs and possible loss of chromosome fragments resulting from translocation heterozygotes (e.g., A7/C6)

**Table 2:** Summary of homologous and non-homologous chromosome pairing in the *Brassica* B genome of a near allo-hexaploid hybrid from assessment of recombination breakpoints in 71 microspore-derived progeny

	B1	B2	B3^a^	B4	B5	B6	B7^a^	B8
homologous	99 %	97 %	97 %	75 %	97 %	100 %	86 %	100 %
non-homologous	1 %	3 %	3 %	25 %	3 %	0 %	14 %	0 %

^a^Univalent inheritance assessed rather than homologous pairing

**Fig. 5:**
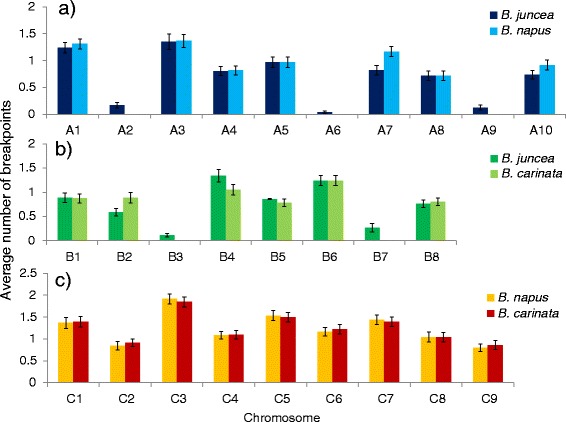
Average number of chromosome recombination breakpoints observed per (**a**) A-genome chromatid, (**b**) B-genome chromatid and (**c**) C-genome chromatid of a near-allohexaploid hybrid, as assessed by marker genotyping of a population of 71 microspore-derived progeny

The average number of recombination breakpoints per chromosome was 0.84 in the A genome, 0.84 in the B genome and 1.25 in the C genome (Fig. 5). Counting only chromosomes with homologous partners, recombination breakpoints per chromosome averaged 0.99 in the A genome and 0.94 in the B genome. Using both measures, the A and B genomes were similar to each other in number of breakpoints per chromosome but significantly different to the C genome (Student’s *t*-test, *p* < 0.05).

### DNA content of MD progeny as estimated by flow cytometry

**Fig. 6:**
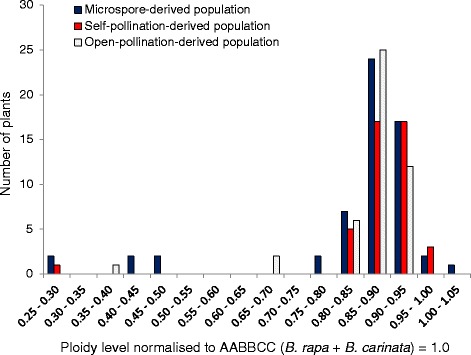
DNA ploidy level as estimated by flow cytometry in three populations derived from microspore culture, self-pollination and open-pollination of the same trigenomic hexaploid hybrid resulting from the cross (*B. napus* × *B. carinata*) × *B. juncea*. Only progeny with consistent DNA content between multiple samples are shown. The Genetics Society of America has granted permission for re-publication of the data from the self-pollinated population for the purposes of comparison: original data published in Mason et al. [25]

DNA ploidy levels in the MD population were inferred from relative DNA content, and ranged from 0.26 to 1.0 of the expected DNA content for a 2n = AABBCC = 54 allohexaploid (Fig. 6). MD progeny with consistent ploidy results fell into two groups: putatively doubled chromosome number (hereafter referred to as “2n”) (53 plants including the unreduced gamete-derived progeny, ploidy range (relative DNA content) 0.76–1.0 and undoubled chromosome number (hereafter referred to as “n”) (6 plants, ploidy range 0.26–0.46) (Fig. 6). Of the 41 MD progeny which were sampled 2–4 times, 12 progeny had > 40 % variation between readings, suggestive of chimeric tissue (both “2n” and “n” chromosome number in sectors of the same plant) (Additional file 5: Table S4). The percentage variation between the maximum and minimum readings for each of the *B. rapa*, *B. napus* and *B. carinata* samples was 2–4 % (Additional file 5: Table S4). However, while some MD, SP and OP plants fell into the 0–5 % variation range, variation within the 5–37 % range was also observed for some plants and in the near-allohexaploid hybrid controls (Additional file 5: Table S4).

### Correlations of chromosome transmission with fertility and ploidy in the MD progeny

As described above, the near-allohexaploid hybrid contained univalent chromosomes for A2, A6, A9, B3 and B7. Of these, only the presence of chromosome A6 was positively correlated with higher self-pollinated seed set (*p* = 0.04) and pollen viability (*p* = 0.03) (Additional file 5: Table S4). Inheritance of parent alleles located on bivalent chromosomes was not significantly associated with fertility (seed set or pollen viability) or ploidy increase across the population using a mixed linear modelling approach after multiple testing corrections. Suggestive correlations (uncorrected *p*-value < 0.05) were found between self-pollinated seed set and marker alleles on the bottom of chromosome A5, top of A6 and top of A3; between pollen viability and the top of chromosome C6, bottom of C7, A3 and B5 and between ploidy status and markers mid-C3, mid-C6, mid-A10 and mid-C7.

### Contrasting fertility of MD, SP and OP progeny

**Fig. 7:**
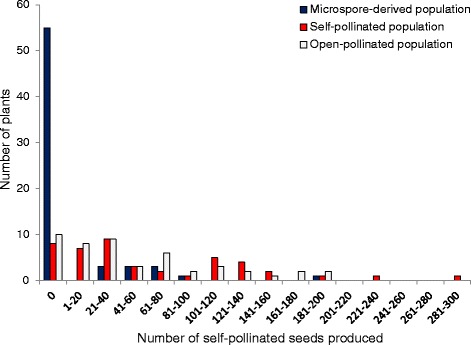
Self-pollinated seed production in three populations derived from microspore culture, self-pollination and open-pollination of the same trigenomic hexaploid hybrid resulting from the cross (*B. napus* × *B. carinata*) × *B. juncea.* The Genetics Society of America has granted permission for re-publication of the data from the self-pollinated population for the purposes of comparison: original data published in Mason et al. [25]

We investigated whether microspore culture yielded progeny with different fertility than self-pollinated (SP) or open-pollinated (OP) progeny. Average seed production per MD individual was 40, which was significantly lower (*p* < 0.05; Student’s *t*-test) than average seed production in SP progeny (78) and OP progeny (68). Of 71 MD plants, 51 (73 %) produced no seed at all (Fig. 7, Additional file 5: Table S4), including 5/6 “n” MD plants. Of the “2n” MD progeny only 30 % (16/53) produced seed, setting 1–198 seeds (40 on average; Fig. 7; Additional file 5: Table S4). In comparison 79 % of OP plants (38/48) and 81 % of SP plants (36/44) set at least 1 self-pollinated seed.

**Fig. 8:**
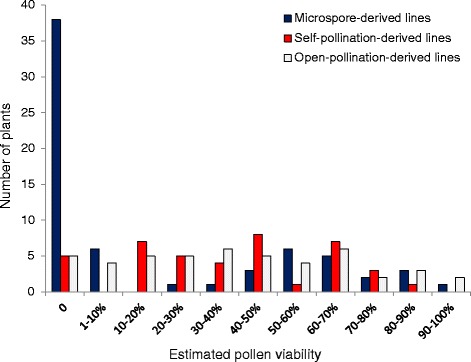
Pollen viability estimates in three populations derived from microspore culture, self-pollination and open-pollination of the same trigenomic hexaploid hybrid resulting from the cross (*B. napus* × *B. carinata*) × *B. juncea.* The Genetics Society of America has granted permission for re-publication of the data from the self-pollinated population for the purposes of comparison: original data published in Mason et al. [25]

Pollen viability ranged from 0 to 91 % in the MD population (Fig. 8) with 58 % of the assayed MD plants being male-sterile (38/66). Similar pollen viability ranges were observed in the SP (0–84 %) and OP (0–94 %) populations, but only 12.5 % (5/40) and 11 % (5/46) of individuals in the SP and OP populations respectively were male-sterile (Fig. 8). Significant correlations (*p* < 0.01) between pollen viability and seed set were present across all individuals and within each of the three populations (Additional file 4: Figure S4, r^2^ = 0.25 for all samples). Fertility as measured by either pollen viability or seed set was not significantly associated with DNA ploidy level in any population (*p* > 0.05), with the exception of a weakly significant association (*p* = 0.043, r^2^ = 0.07) between ploidy and pollen viability in the MD population.

## Discussion

By tracking SNP allele inheritance along individual chromatids in the MD population, we could identify chromosome rearrangements and predict the frequency of homologous and non-homologous recombination events during meiosis in the near-allohexaploid hybrid. Although a low level of non-homologous recombination between each of the A, B and C genomes was identified, A^j^-A^n^, B^j^-B^c^ and C^n^-C^c^ homologous chromosomes recombined and segregated with high fidelity in the near-allohexaploid hybrid, with mostly regular allele transmission and minimal non-homologous interactions. Examples of irregular meiosis were almost entirely related to illegitimate pairing between homoeologues, but occasionally due to putative univalent inheritance or interactions between other non-homologous chromosome regions. The majority of non-homologous associations were predicted to involve both pre-existing translocation events and univalent chromosomes. Use of an MD population allowed clear, detailed inferences about crossover frequency and gamete production in the trigenomic hybrid.

Recently, significant progress has been made in the *Brassica* model in determining how crossover formation during meiosis is regulated, and what role this plays in subsequent chromosome rearrangements and meiotic stability [13]. At least two different but complementary gene pathways are responsible for regulating the location and frequency of crossover formation and resolution during meiosis [28]. However, it appears that crossover frequency is at least in part regulated by cell chromosome number: the number of crossovers observed in series of hybrids and haploids is known to increase with increasing numbers of univalent chromosomes [14, 29, 30]. Recent work has also shown that retention or loss of particular individual chromosomes, such as C9, can also affect crossover frequency [31]. Our results suggest that crossover frequency was higher per chromatid in the C genome of the *B. napus* × *B. carinata* CCAB hybrid (Fig. 2; average 3.0 chromatid breakpoints detected per chromatid) than in the allohexaploid hybrid (1.25 breakpoints) and in synthetic *B. napus* (1.0 crossovers per recombining chromosome; [14]). This confirms previous speculation regarding increased crossover frequency in diploid genomes in this and similar hybrid types containing high numbers of additional univalent chromosomes [19, 30] and matches observations in allotriploid (AAC) hybrids relative to diploids (AA) and allotetraploids (AACC) [14]. In addition, the higher frequency of recombination in the C genome (1.2 breakpoints/chromatid) compared to the A and B genomes (0.84 breakpoints/chromatid) in the near-allohexaploid hybrid (Fig. 4) may suggest that increased sequence similarity between C^n/c^ chromatids resulting from the previous C^n^-C^c^ recombination events facilitates crossover formation, relative to more distinct chromatids in unrecombined B^j^-B^c^ and A^j^-A^n^ chromosome pairs.

Bias towards inheritance of univalent chromosomes appeared to be regulated somewhat differently than bias towards inheritance of alleles from homologous chromosome pairs. In this study, several univalent chromosomes showed strong bias towards retention or loss in the population, although few strong effects were observed for chromosomes present as homologous pairs. In particular, B^j^7 was preferentially lost, whereas A^j^9 and B^j^3 were preferentially retained (Additional file 4: Figures S1 and S2), putatively through selective pressure for or against particular alleles present on those chromosomes. This is not entirely expected due to the high degree of genomic redundancy offered by the presence of three related genomes in these hybrids, but could be related to disruptions to gene dosage balance rather than to absolute loss or gain of effect [32]. Interestingly, although there was no bias towards retention or loss of chromosome A^j^6 in the MD population, presence of this chromosome was positively associated with both seed set and pollen viability (Additional file 4: Figure S4). No other univalent chromosome had an effect on fertility, despite the extreme bias towards inheritance of some chromosomes, suggesting that some chromosomes or chromosomal loci may be more important for somatic viability but not fertility and vice versa. Although B genome inheritance was not assessed in the self-pollinated population analysed previously [25], bias towards inheritance of chromosome A^j^9 was common to both populations. Hence, A^j^9 may contain genetic factors related to hybrid embryo viability.

The A7-C6 translocation is a known feature of many *B. napus* genotypes [33], and so it was not surprising to observe this in the *B. napus* cross parent of our study (and in our previous work with a related population [25]). The presence of a large scale duplication of loci on chromosome A10 was also observed in this *B. napus* parent (see [25]). We were unable to identify a deleted genomic region in the C genome corresponding to this duplication, but possibly a region of homoeologue C9 was deleted. An approximately 2 Mbp duplication of part of chromosome C4 was also detected and determined to have arisen from a non-reciprocal translocation with A4, probably during meiosis in the *B. napus* × *B. carinata* hybrid. Interestingly, the two pre-existing genomic features in *B. napus* (the A7-C6 translocation and the A10 duplication) were responsible for the vast majority of homologous pairing disruption observed in our study, similarly to the sister population derived from self-pollination events [25]. The genomic architecture of *B. juncea*, *B. carinata* and *B. napus* appears to be otherwise remarkably conserved (Additional file 4: Figures S1-S3), considering the widely spaced timing of the independent speciation events which gave rise to each allotetraploid species [34].

On average, MD progeny fertility was much lower than that of the SP and OP populations (Figs. 7 and 8), despite the fact that most MD progeny had doubled chromosome number during microspore culture (Fig. 6). This may suggest that non-viable chromosome rearrangement events have been fixed in the sterile plants, or that accumulation of more duplication and deletion events present in two copies rather than one is more likely to result in disruption of alleles related to fertility [35]. However, this effect may also be partially attributable to the additional stress exerted on this material by the microspore culture process. At least 12 of the MD plants were chimeric, containing sectors of doubled and undoubled tissue (Additional file 5: Table S4). While this is not unexpected in *Brassica* progeny derived from microspore culture [36], a high level of variation between readings was observed in most higher-ploidy MD progeny, but not in established species controls (Additional file 5: Table S4). Furthermore, deviations from the expected two copies of chromosome pairs were observed in many putatively “2n” MD progeny (Additional file 4: Figure S5, Additional file 5: Table S4), and cannot be explained by any known meiotic mechanism. A likely source of these anomalies is mitotic instability that may have occurred either as a result of the hybrid status of these progeny, response to colchicine treatment or to the microspore culture processes, or a combination of these factors. This finding may inspire further investigations of the mechanisms governing mitotic instability in microspore culture, an important tool in *Brassica* breeding and genetics research.

Assessment of meiotic behaviour was greatly assisted in this study by the availability of a population of microspore-derived progeny, which sampled individual male gametes from the near-allohexaploid hybrid. Such technology is currently available in few plant species. However, as next-generation sequencing techniques advance, single-cell sequencing may allow gametes (pollen, ovules) to be isolated and sequenced directly without the medium of microspore culture to develop embryos and plants, broadening the species applicability of this analysis approach. High-throughput molecular karyotyping approaches are being increasingly available to answer biological questions. The use of SNP chip arrays may rapidly become superseded by genotyping-by-sequencing [37]. However, the analytical power provided by the SNP chip arrays over other molecular marker types, combined with developed pipelines for data analysis [27] have allowed us to determine meiotic behaviour in this allohexaploid rapidly and effectively.

## Conclusions

SNP marker segregation patterns in the microspore-derived (MD) population, each of which result from single male meiosis in a near-allohexaploid *Brassica* F1 hybrid, allowed us to infer detailed meiotic behaviour in this hybrid. Crossover frequencies per chromosome in the allohexaploid were determined to be similar to frequencies expected in established *Brassica* species, suggesting that higher chromosome numbers are not disruptive to cellular regulation of crossover events. The MD population was found to have lower average fertility and highly variable DNA ploidy levels compared to SP and OP populations grown under the same experimental conditions [25]. Production of a new, stable and fertile allohexaploid *Brassica* crop species through genomic breeding, bringing together all the genetic diversity of the cultivated *Brassica* species and concentrating allelic heterosis, offers a pathway of significant interest for crop geneticists.

## Methods

### Experimental material

A four-generation pedigree was employed in this experiment (Fig. 1). Generation 1 and Generation 2 involved intercrossing three allotetraploid *Brassica* species resulting in a near-allohexaploid hybrid (2n = 49; Generation 3), named “N1C1.J1-1” by Mason et al. [24]. This crossing approach relied on the phenomenon of unreduced gamete formation in the CCAB hybrid to produce gametes with a CCAB genome composition (Generation 2), rather than the 9C + 0-10A + 0-8B chromosome complement expected for a reduced gamete from this hybrid type. The parental genotypes used were: doubled-haploid lines of *Brassica napus* (“Surpass400-024DH”) and *B. carinata* (“195923.3.2_01DH”); and the *B. juncea* experimental breeding line “JN9-04” [24]. The near-allohexaploid hybrid was subjected to microspore culture according to protocols of Takahira et al. [36] and Cousin and Nelson [26] (using the chromosome doubling agent, colchicine), and resulted in a population of 75 microspore-derived (MD) progeny (Generation 4). After removal from tissue culture, the MD population was grown to maturity in a controlled environment room (16 h photoperiod with a light intensity of 200 μmol m^−2^ s^−1^ at 18 °/10 °C day/night) at The University of Western Australia.

A population of 50 seeds derived from self-pollination (SP) and 50 seeds derived from open-pollination (OP) of the same near-allohexaploid hybrid were planted approximately 5 weeks after removal of the MD progeny to the controlled environment room. Open-pollinated seeds were collected from racemes of the hybrid growing in proximity to other experimental hybrids and allotetraploid *Brassica* lines, and so could have resulted from either cross-pollination or self-pollination. A total of 44 SP seeds and 48 OP seeds germinated, after which all individuals in the three populations were grown to full maturity and self-seed set under the same environmental conditions. DNA ploidy level (flow cytometry), pollen viability and self-pollinated seed set data for the plants derived from self-pollination of the near-allohexaploid hybrid were previously reported in Mason et al. [25].

### DNA extractions, flow cytometry and fertility

DNA was extracted from leaf tissue of experimental progeny, parents and hybrids using the Illustra Nucleon Phytopure Genomic DNA Extraction kits (GE Healthcare) according to manufacturer’s instructions. Flow cytometry was carried out according to protocols detailed in Mason et al. [25]. Each MD plant was sampled 1–4 times: 34 plants were sampled once, 22 plants were sampled twice, 13 plants were sampled 3 times and 6 plants were sampled 4 times. SP plants were also sampled 1–4 times: 18 plants were sampled once, 15 plants were sampled twice, 10 plants were sampled 3 times and one plant was sampled 4 times. OP plants were sampled once (26 plants) or twice (21 plants), and data were not obtained for one plant. Plants from parental *B. napus* and *B. carinata* genotypes were each sampled four times and *B. rapa* “Chiifu” was sampled five times. A propagated cutting of the near-allohexaploid hybrid plant was sampled four times. In order to assess technical variability in DNA ploidy estimates, three different cuttings from another trigenomic hybrid resulting from the same cross (plant “A2” in Mason et al. [24]) were each sampled 3–4 times. Lettuce (*Lactuca sativa*) was used as an internal control and samples were normalised to 1.0 = *B. rapa* + *B. carinata* ploidy levels (2n = ~AABBCC). Self-pollination of all plants was encouraged by enclosing racemes in microperforated plastic bread bags, and pollen viability was estimated using 1 % acetocarmine stainability according to methods detailed in Mason et al. [25].

### Molecular markers

DNA from all parents, hybrids and MD progeny in the four-generation pedigree were genotyped using Illumina *Brassica* 60K SNP chips (Illumina Inc., San Diego, USA), according to manufacturer’s instructions. Chips were scanned using a HiScanSQ (Illumina Inc). SNP marker data were visualised using Genome Studio V2011.1 (Illumina Inc, San Diego, USA). SNP markers that were polymorphic between the *B. napus* and *B. carinata* parents (C genome) and between the *B. napus* and *B. juncea* parents (A genome) were retained, and SNP marker clustering manually checked. SNP markers were ordered within their respective chromosomes with reference to the sequenced A genome of *B. rapa* [38] and C genome of *B. oleracea* [39] and misplaced contigs relocated by manual inspection of haplotype continuity.

Copy number variation for the subset of SNPs used in this study was analyzed based on the log R ratio (LRR) calculated in Genome Studio, in line with a previously established and validated methodology for detection of copy number variation using SNP genotyping arrays [40, 41]. The LRR, which defines the observed fluorescence intensity relative to the expected fluorescence intensity (derived from the average of all SNPs in that genotype cluster) transformed on log scale, and the B allele frequencies (BAF), which measure the relative contribution of the B alleles to the combined A and B allele signals, were exported from Genome Studio and plotted for each SNP along their putative chromosomal positions in the *Brassica napus* genome (Additional file 4: Figure S5) [42]. LRRs which cluster around 0 are assumed to have no copy number variation, i.e., a copy number of 2 in most species and in somatically doubled MD progeny. The BAF values mainly cluster around 0, 0.5 and 1, corresponding to the genotypes AA, AB and BB (Additional file 4: Figure S5).

Molecular karyotyping of B-genome chromosomes was performed using SSRs which were selected after first screening for polymorphism between the *B. carinata* and *B. juncea* B-genome parents. These markers comprised: (1) a set of 48 B genome-specific SSR primers of known genomic location [43, 44] provided by Agriculture and Agri-Food Canada (AAFC) (Isobel Parkin, personal communication) and previously screened across a panel of U Triangle species samples [35]; (2) 115 publically available markers developed for the *Brassica* A and C genomes, but which also amplified B-genome alleles (Isobel Parkin, personal communication; http://aafc-aac.usask.ca/BrassicaMAST/). Polymorphic SSR markers were then used to genotype the MD population along with hybrid and parental controls. B-genome markers with known genome locations from set 1 were used to place B-genome alleles with unknown locations (set 2) on linkage groups (Additional file 1: Table S1) using a linkage mapping approach. Fragment analysis was performed using an AB3730xl DNA sequencer (Applied Biosystems, Scoresby, Victoria) with fluorescently-labelled primers, or via agarose gel electrophoresis [45]. The resulting B-genome linkage groups were named B1 to B8 following the numbering convention established by Lagercrantz and Lydiate [46].

### Statistical analyses

Pearson’s *χ*^2^ test was used to detect deviation from 1:1 segregation ratios (AA: BB genotype calls) expected for an MD population. Chromosome segments were determined to be absent if more than five contiguous SNPs had no call (NC) scores within a single individual but were present in other members of the MD population. Linear models in the R version 3.0.0 base package (The R project for statistical computing) were used to calculate F statistics, r^2^ correlation coefficients and *p*-values for the relationship between DNA ploidy level, seed-set and pollen viability in and across each of the three populations (function lm()). Generalised linear models (function glm()) using the binomial distribution were used to assess the impact of presence/absence of individual chromosomes and particular translocation, deletion and duplication events on self-pollinated seed set and pollen viability.

Heatmaps were generated using the “heatmap.2” function in the gplots package of R version 3.0.0 and formatted using Microsoft PowerPoint 2010 (Microsoft Corporation). Redundant SNP marker alleles were reduced to a single representative per “marker bin” using an R script that compared allele inheritance across the population and removed each progeny that matched a previous progeny exactly, treating missing values as wild cards [27]. Fisher’s exact test for count data in R was used on count summaries generated for each marker allele pair: both alleles present, neither allele present, first allele present or second allele present. Expected value tables to account for unequal allele frequencies were produced using 2 × 2 contingency tables.

### Association analysis

Association analysis was performed using a compressed mixed linear modelling approach ([47]) implemented in the “GAPIT” package of R ([48]), in order to determine if any genomic regions were significantly associated with phenotypic traits. R function “na.gam.replace” in the “gam” package was used to replace missing genotypic values with SNP marker averages for the population. Pollen viability, seed set and ploidy status were used as phenotypic input variables.

## Availability of supporting data

The data sets supporting the results of this article are included within the article and its additional files.

## Additional files

Additional file 1: Table S1. Segregating SNP loci in the A and C genomes of an allohexaploid *Brassica* ((*B. napus* × *B. carinata*) × *B. juncea*) microspore-derived (MD) population. Additional file 2: Table S2. Segregating SSR loci in the B genome of an allohexaploid *Brassica* ((*B. napus × B. carinata*) *× B. juncea*) microspore-derived (MD) population. Additional file 3: Table S3. Allele presence and absence in the A and C genomes in an allohexaploid *Brassica* ((*B. napus × B. carinata*) *× B. juncea*) microspore-derived (MD) population. “NA” indicates no amplification or missing data; “Aj” the *B. juncea* A genome, “An” the *B. napus* A genome, “Bc” the *B. carinata* B genome, “Bj” the *B. juncea* B genome, “Cc” the *B. carinata* genome and “Cn” the *B. napus* genome. Additional file 4: Figure S1. Transmission frequencies of *B. juncea* and *B. napus* A-genome SNP marker alleles from a near-allohexaploid hybrid to a population of 71 microspore-derived progeny. Horizontal dotted lines show thresholds for significant deviation from 50 % inheritance (*p* < 0.05, Pearson’s chi-squared test). * Chromosomes A^n^2, A^n^6 and A^n^9 were absent in the near-allohexaploid hybrid. **Figure S2.** Transmission frequencies of *B. juncea* and *B. carinata* B-genome SSR marker alleles from a near-allohexaploid hybrid to a population of 71 microspore-derived progeny. Horizontal dotted lines show thresholds for significant deviation from 50 % inheritance (*p* < 0.05, Pearson’s chi-squared test). * Chromosomes B^c^3 and B^c^7 were absent in the near-allohexaploid hybrid. **Figure S3.** Transmission frequencies of *B. napus* and *B. carinata* C-genome SNP marker alleles from a near-allohexaploid hybrid to a population of 71 microspore-derived progeny. Horizontal dotted lines indicate significant deviation from 50 % inheritance (*p* < 0.05, Pearson’s chi-squared test). **Figure S4.** The relationship between pollen viability estimates and self-pollinated seed production in three populations derived from microspore culture, self-pollination and open-pollination of the same trigenomic hexaploid hybrid resulting from the cross (*B. napus* × *B. carinata*) × *B. juncea*. **Figure S5.** Copy number variation (CNV) for A and C genome SNP alleles in A) *B. carinata*, b) an allohexaploid hybrid and C) the microspore-derived progeny MD_042. In the upper plots, for each SNP the Log R Ratio is plotted according to chromosomal position in the *Brassica napus* genome. Dots (SNPs) above the line indicate a higher copy number for those SNPs relative to the other SNPs and dots below the line indicate a lower copy number of those SNPs relative to the other SNPs. The lower plots show the corresponding B allele frequencies which assign the relative contribution of each B allele to all signals from both the A to B alleles, where a ratio of 0.5 indicates heterozygosity (AB) and 0 or 1 homozygosity (presence or absence of the B allele). A) *B. carinata* - absence of all A-genome chromosomes and presence of two identical copies of each C-genome chromosome. B) Allohexaploid hybrid - one copy of chromosomes A2, A6 and A9 and two copies of all other chromosomes. C) Example microspore-derived progeny MD_042 from the near-allohexaploid hybrid - absence of chromosomes A2, A6 and part of C6, an extra copy of chromosome A5 and a duplication of part of chromosome A7. Additional file 5: Table S4. Pollen viability, self-pollinated seed set and DNA ploidy level as estimated by flow cytometry in three populations derived from microspore culture (MD), self-pollination (SP) and open-pollination (OP) of the same trigenomic hexaploid hybrid resulting from the cross (*B. napus* × *B. carinata*) × *B. juncea*, along with parent and trigenomic hybrid (5–6x three genome aneuploid) controls.

## Abbreviations

MD: Microspore-derived
OP: Open-pollinated
SNP: Single Nucleotide Polymorphism
SP: Self-pollinated
SSR: Simple Sequence Repeat
LRR: Log R Ratio

## References

[1] Dixon GR, Atherton J, Rees H (2007). Vegetable Brassicas and related crucifers. Crop production science in horticulture series.

[2] U N (1935). Genome-analysis in *Brassica* with special reference to the experimental formation of *B. napus* and peculiar mode of fertilization. Jpn J Botany.

[3] Morinaga T (1934). Interspecific hybridisation in *Brassica* VI: the cytology of F_1_ hybrids of *B. juncea* and *B. nigra*. Cytologia.

[4] Chen S, Nelson MN, Chèvre A-M, Jenczewski E, Li Z, Mason AS, Meng J, Plummer JA, Pradhan A, Siddique KH (2011). Trigenomic bridges for *Brassica* improvement. Crit Rev Plant Sci.

[5] Snowdon R (2007). Cytogenetics and genome analysis in *Brassica* crops. Chromosom Res.

[6] Cowling WA (2007). Genetic diversity in Australian canola and implications for crop breeding for changing future environments. Field Crop Res.

[7] Snowdon RJ, Luy FLI (2012). Potential to improve oilseed rape and canola breeding in the genomics era. Plant Breed.

[8] Szadkowski E, Eber F, Huteau V, Lodé M, Huneau C, Belcram H, Coriton O, Manzanares-Dauleux MJ, Delourme R, King GJ (2010). The first meiosis of resynthesized *Brassica napus*, a genome blender. New Phytol.

[9] Song KM, Lu P, Tang KL, Osborn TC (1995). Rapid genome change in synthetic polyploids of *Brassica* and its implications for polyploid evolution. Proc Natl Acad Sci U S A.

[10] Tian E, Jiang Y, Chen L, Zou J, Liu F, Meng J (2010). Synthesis of a *Brassica* trigenomic allohexaploid (*B. carinata* × *B. rapa*) de novo and its stability in subsequent generations. Theor Appl Genet.

[11] Iwasa S (1964). Cytogenetic studies on the artificially raised trigenomic hexaploid hybrid forms in the genus *Brassica*. J Faculty Agri, Kyushu Univ.

[12] Nicolas SD, Le Mignon G, Eber F, Coriton O, Monod H, Clouet V, Huteau V, Lostanlen A, Delourme R, Chalhoub B (2007). Homeologous recombination plays a major role in chromosome rearrangements that occur during meiosis of *Brassica napus* haploids. Genetics.

[13] Nicolas SD, Monod H, Eber F, Chèvre AM, Jenczewski E (2012). Non-random distribution of extensive chromosome rearrangements in *Brassica napus* depends on genome organization. Plant J.

[14] Leflon M, Grandont L, Eber F, Huteau V, Coriton O, Chelysheva L, Jenczewski E, Chevre AM (2010). Crossovers get a boost in *Brassica* allotriploid and allotetraploid hybrids. Plant Cell.

[15] Xiong ZY, Gaeta RT, Pires JC (2011). Homoeologous shuffling and chromosome compensation maintain genome balance in resynthesized allopolyploid *Brassica napus*. Proc Natl Acad Sci U S A.

[16] Gaeta RT, Pires JC, Iniguez-Luy F, Leon E, Osborn TC (2007). Genomic changes in resynthesized *Brassica napus* and their effect on gene expression and phenotype. Plant Cell.

[17] Geng XX, Chen S, Astarini IA, Yan GJ, Tian E, Meng J, Li ZY, Ge XH, Nelson MN, Mason AS (2013). Doubled haploids of novel trigenomic *Brassica* derived from various interspecific crosses. Plant Cell Tiss Org.

[18] Harberd DJ, McArthur ED, Tsunoda S, Hinata K, Gomez-Campo C (1980). Meiotic analysis of some species and genus hybrids in the Brassiceae. Brassica crops and wild allies: biology and breeding.

[19] Nicolas SD, Leflon M, Monod H, Eber F, Coriton O, Huteau V, Chèvre AM, Jenczewski E (2009). Genetic regulation of meiotic cross-overs between related genomes in *Brassica napus* haploids and hybrids. Plant Cell.

[20] Parkin IAP, Sharpe AG, Lydiate DJ (2003). Patterns of genome duplication within the *Brassica napus* genome. Genome.

[21] Jenczewski E, Eber F, Grimaud A, Huet S, Lucas MO, Monod H, Chèvre AM (2003). *PrBn*, a major gene controlling homeologous pairing in oilseed rape (*Brassica napus*) haploids. Genetics.

[22] Liu Z, Adamczyk K, Manzanares-Dauleux M, Eber F, Lucas M-O, Delourme R, Chèvre AM, Jenczewski E E (2006). Mapping *PrBn* and other quantitative trait loci responsible for the control of homeologous chromosome pairing in oilseed rape (*Brassica napus* L.) haploids. Genetics.

[23] Griffiths S, Sharp R, Foote TN, Bertin I, Wanous M, Reader S, Colas I, Moore G (2006). Molecular characterization of *Ph1* as a major chromosome pairing locus in polyploid wheat. Nature.

[24] Mason AS, Yan GJ, Cowling WA, Nelson MN (2012). A new method for producing allohexaploid *Brassica* through unreduced gametes. Euphytica.

[25] Mason AS, Nelson MN, Takahira J, Cowling WA, Moreira Alves G, Chaudhuri A, Chen N, Ragu ME, Dalton-Morgan J, Coriton O (2014). The fate of chromosomes and alleles in an allohexaploid *Brassica* population. Genetics.

[26] Cousin A, Nelson M (2009). Twinned microspore-derived embryos of canola (*Brassica napus* L.) are genetically identical. Plant Cell Rep.

[27] Mason AS, Batley J, Bayer PE, Hayward A, Cowling WA, Nelson MN (2014). High-resolution molecular karyotyping uncovers pairing between ancestrally related *Brassica* chromosomes. New Phytol.

[28] Crismani W, Girard C, Froger N, Pradillo M, Santos JL, Chelysheva L, Copenhaver GP, Horlow C, Mercier R (2012). FANCM limits meiotic crossovers. Science.

[29] Nagpal R, Raina SN, Sodhi YS, Mukhopadhyay A, Arumugam N, Pradhan AK, Pental D D (1996). Transfer of *Brassica tournefortii* (TT) genes to allotetraploid oilseed *Brassica* species (*B. juncea* AABB, *B. napus* AACC, *B. carinata* BBCC): homoeologous pairing is more pronounced in the three-genome hybrids (TACC, TBAA, TCAA, TCBB) as compared to allodiploids (TA, TB, TC). Theor Appl Genet.

[30] Nicolas SD, Leflon M, Liu Z, Eber F, Chelysheva L, Coriton O, Chèvre AM, Jenczewski E (2008). Chromosome ‘speed dating’ during meiosis of polyploid *Brassica* hybrids and haploids. Cytogenet Genome Res.

[31] Suay L, Zhang D, Eber F, Jouy H, Lodé M, Huteau V, Coriton O, Szadkowski E, Leflon M, Martin OC (2014). Crossover rate between homologous chromosomes and interference are regulated by the addition of specific unpaired chromosomes in *Brassica*. New Phytol.

[32] Bekaert M, Edger PP, Pires JC, Conant GC (2011). Two-phase resolution of polyploidy in the *Arabidopsis* metabolic network gives rise to relative and absolute dosage constraints. Plant Cell.

[33] Osborn TC, Butrulle DV, Sharpe AG, Pickering KJ, Parkin IA, Parker JS, Lydiate DJ (2003). Detection and effects of a homeologous reciprocal transposition in *Brassica napus*. Genetics.

[34] Palmer JD, Shields CR, Cohen DB, Orton TJ (1983). Chloroplast DNA evolution and the origin of amphidiploid *Brassica* species. Theor Appl Genet.

[35] Mason AS, Nelson MN, Castello M-C, Yan G, Cowling WA (2011). Genotypic effects on the frequency of homoeologous and homologous recombination in *Brassica napus* × *B. carinata* hybrids. Theor Appl Genet.

[36] Takahira J, Cousin A, Nelson MN, Cowling WA (2011). Improvement in efficiency of microspore culture to produce doubled haploid canola (*Brassica napus* L.) by flow cytometry. Plant Cell Tiss Org.

[37] Bayer PE, Ruperao P, Mason AS, Stiller J, Chan C-KK, Hayashi S, et al. High-resolution skim genotyping by sequencing reveals the distribution of crossovers and gene conversions in *Cicer arietinum* and *Brassica napus*. Theor. Appl. Genet. 2015, In Press. doi: 10.1007/s00122-00015-02488-y. 10.1007/s00122-015-2488-y 25754422

[38] Wang XW, Wang HZ, Wang J, Sun RF, Wu J, Liu SY, Bai YQ, Mun JH, Bancroft I, Cheng F (2011). The genome of the mesopolyploid crop species *Brassica rapa*. Nat Genet.

[39] Parkin IA, Koh C, Tang H, Robinson SJ, Kagale S, Clarke WE, Town CD, Nixon J, Krishnakumar V, Bidwell SL (2014). Transcriptome and methylome profiling reveals relics of genome dominance in the mesopolyploid *Brassica oleracea*. Genome Biol.

[40] Nannya Y, Sanada M, Nakazaki K, Hosoya N, Wang LL, Hangaishi A, Kurokawa M, Chiba S, Bailey DK, Kennedy GC (2005). A robust algorithm for copy number detection using high-density oligonucleotide single nucleotide polymorphism genotyping arrays. Cancer Res.

[41] McCarroll SA, Kuruvilla FG, Korn JM, Cawley S, Nemesh J, Wysoker A, Shapero MH, de Bakker PIW, Maller JB, Kirby A (2008). Integrated detection and population-genetic analysis of SNPs and copy number variation. Nat Genet.

[42] Chalhoub B, Denoeud F, Liu SY, Parkin IAP, Tang HB, Wang XY, Chiquet J, Belcram H, Tong CB, Samans B (2014). Early allopolyploid evolution in the post-Neolithic *Brassica napus* oilseed genome. Science.

[43] Navabi ZK, Parkin IA, Pires JC, Xiong Z, Thiagarajah MR, Good AG, Rahman MH (2010). Introgression of B-genome chromosomes in a doubled haploid population of Brassica napus x B. carinata. Genome.

[44] Chen S, Wan ZJ, Nelson MN, Chauhan JS, Redden R, Burton WA, Lin P, Salisbury PA, Fu TD, Cowling WA (2013). Evidence from genome-wide simple sequence repeat markers for a polyphyletic origin and secondary centers of geneticdiversity of *Brassica juncea* in China and India. J Hered.

[45] Nelson MN, Mason AS, Castello MC, Thomson L, Yan GJ, Cowling WA (2009). Microspore culture preferentially selects unreduced (2n) gametes from an interspecific hybrid of *Brassica napus* L. x *Brassica carinata* Braun. Theor Appl Genet.

[46] Lagercrantz U, Lydiate DJ (1995). RFLP mapping in *Brassica nigra* indicates differing recombination rates in male and female meioses. Genome.

[47] Zhang ZW, Ersoz E, Lai CQ, Todhunter RJ, Tiwari HK, Gore MA, Bradbury PJ, Yu JM, Arnett DK, Ordovas JM (2010). Mixed linear model approach adapted for genome-wide association studies. Nat Genet.

[48] Lipka AE, Tian F, Wang Q, Peiffer J, Li M, Bradbury PJ, Gore MA, Buckler ES, Zhang Z (2012). GAPIT: genome association and prediction integrated tool. Bioinformatics.

