# Supplementary material for: Microspore culture reveals complex meiotic behaviour in a trigenomic Brassica hybrid
Source: BMC Plant Biol. 2015 Jul 8;15:173. doi: 10.1186/s12870-015-0555-9 (PMC4493989; doi:10.1186/s12870-015-0555-9)
Supplement: Additional file 4: Figure S1. — Transmission frequencies of B. juncea and B. napus A-genome SNP marker alleles from a near-allohexaploid hybrid to a population of 71 microspore-derived progeny. Horizontal dotted lines show thresholds for significant deviation from 50 % inheritance (p < 0.05, Pearson’s chi-squared test). * Chromosomes An2, An6 and An9 were absent in the near-allohexaploid hybrid. Figure S2. Transmission frequencies of B. juncea and B. carinata B-genome SSR marker alleles from a near-allohexaploid hybrid to a population of 71 microspore-derived progeny. Horizontal dotted lines show thresholds for significant deviation from 50 % inheritance (p < 0.05, Pearson’s chi-squared test). * Chromosomes Bc3 and Bc7 were absent in the near-allohexaploid hybrid. Figure S3. Transmission frequencies of B. napus and B. carinata C-genome SNP marker alleles from a near-allohexaploid hybrid to a population of 71 microspore-derived progeny. Horizontal dotted lines indicate significant deviation from 50 % inheritance (p < 0.05, Pearson’s chi-squared test). Figure S4. The relationship between pollen viability estimates and self-pollinated seed production in three populations derived from microspore culture, self-pollination and open-pollination of the same trigenomic hexaploid hybrid resulting from the cross (B. napus × B. carinata) × B. juncea. Figure S5. Copy number variation (CNV) for A and C genome SNP alleles in A) B. carinata, b) an allohexaploid hybrid and C) the microspore-derived progeny MD_042. In the upper plots, for each SNP the Log R Ratio is plotted according to chromosomal position in the Brassica napus genome. Dots (SNPs) above the line indicate a higher copy number for those SNPs relative to the other SNPs and dots below the line indicate a lower copy number of those SNPs relative to the other SNPs. The lower plots show the corresponding B allele frequencies which assign the relative contribution of each B allele to all signals from both the A to B alleles, where a ratio of 0.5 indicates [file 12870_2015_555_MOESM4_ESM.pptx]

## Slide 1
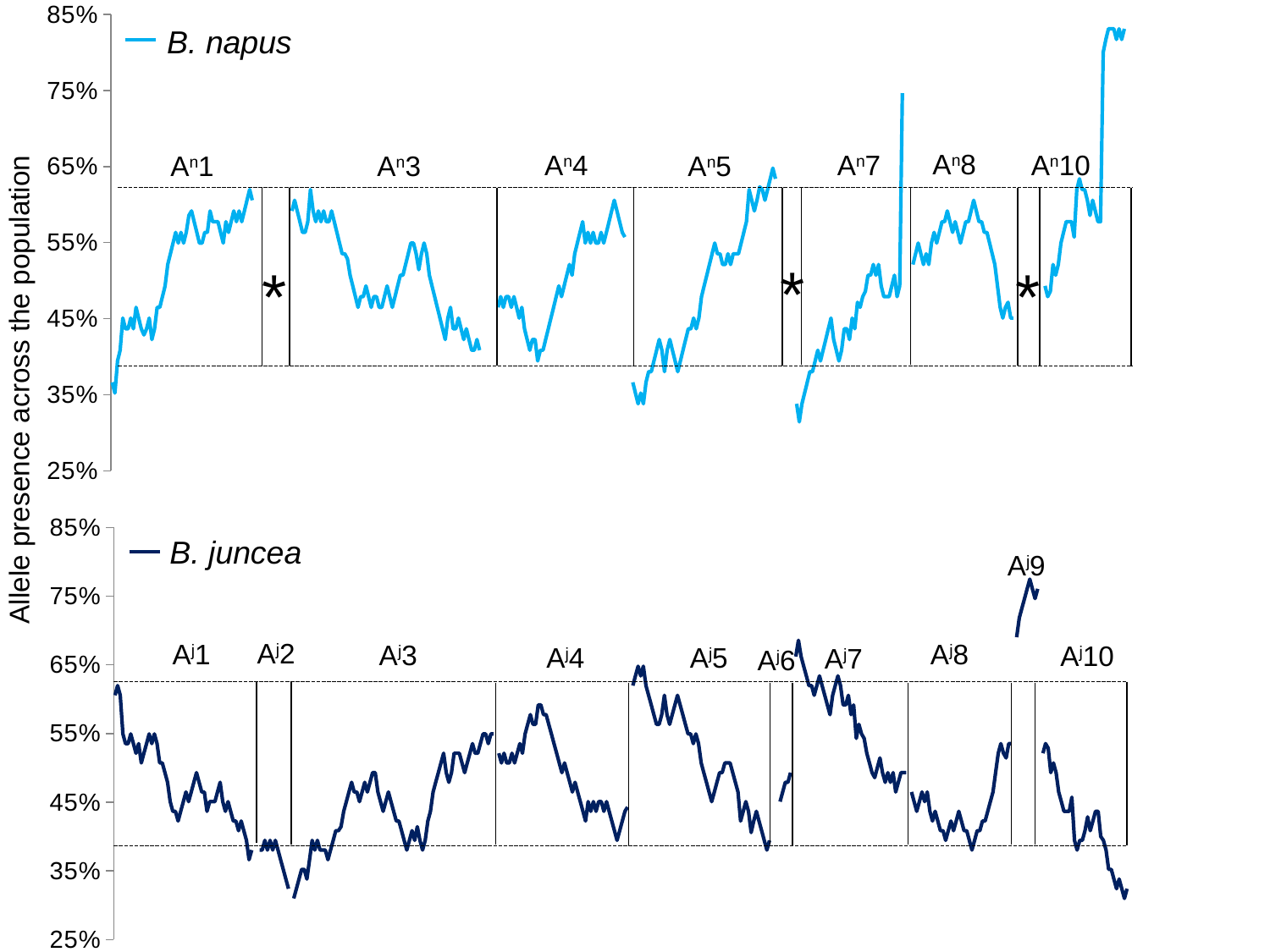

### Chart
| Category | |
|---|---|B. napus
An8
An7
An10
An4
An5
An1
An3
*
*
*
Allele presence across the population
### Chart
| Category | |
|---|---|B. juncea
Aj9
Aj2
Aj8
Aj1
Aj3
Aj10
Aj5
Aj4
Aj7
Aj6
Figure S1: Transmission frequencies of B. juncea and B. napus A-genome SNP marker alleles from a near-allohexaploid hybrid to a population of microspore-derived lines. Horizontal dotted lines show thresholds for significant deviation from 50% inheritance (p<0.05, Pearson’s chi-squared test). * Chromosomes An2, An6 and An9 were absent in the near-allohexaploid hybrid.

## Slide 2
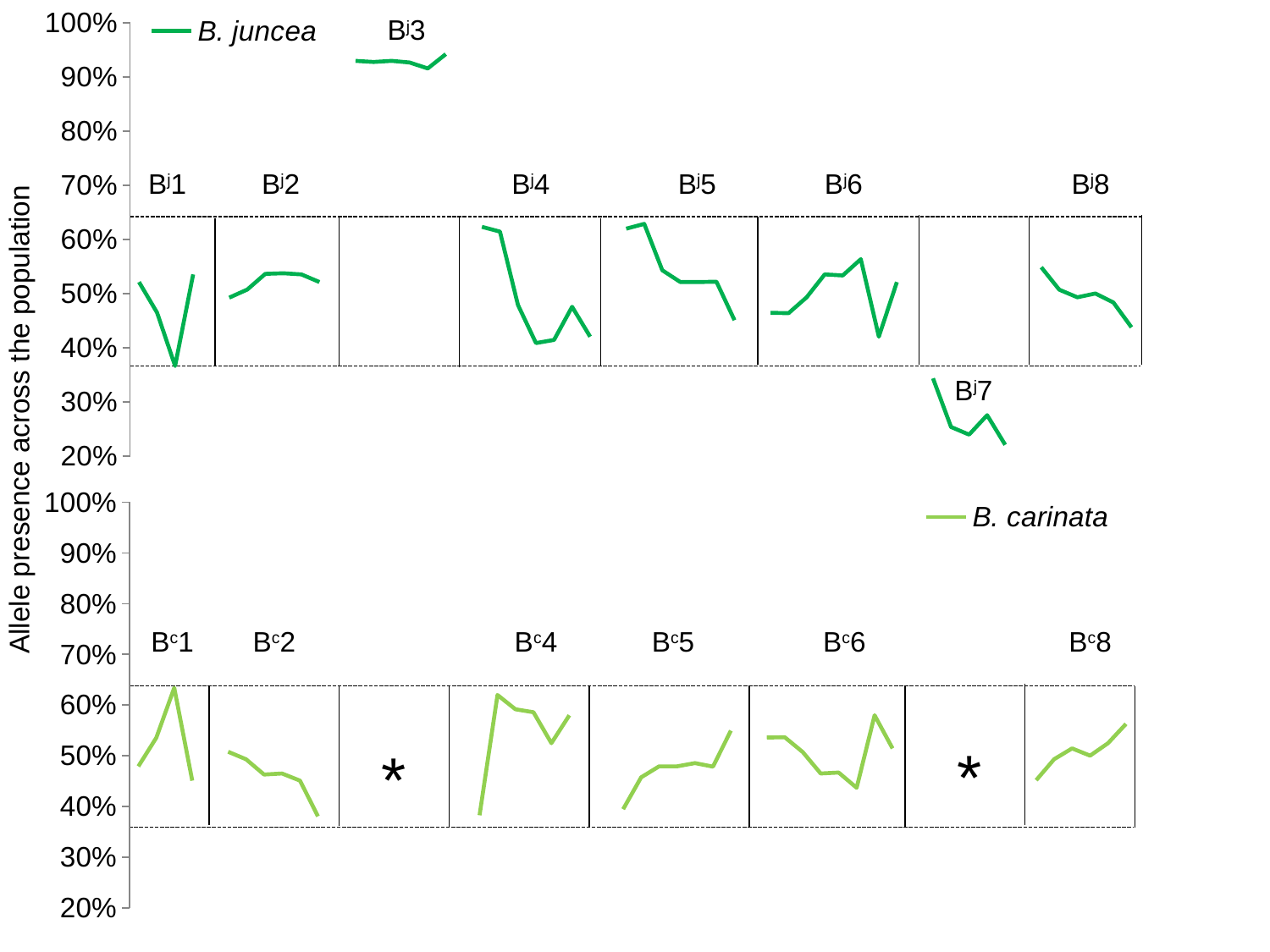

### Chart
| Category | |
|---|---|Bj3
Bj1
Bj2
Bj4
Bj5
Bj6
Bj8
Bj7
Allele presence across the population
Bc1
Bc2
Bc4
Bc5
Bc6
Bc8
*
*
### Chart
| Category | |
|---|---|
| Bc1_01 | 0.4788732394366197 |
| Bc1_02 | 0.5352112676056338 |
| Bc1_03 | 0.6338028169014085 |
| Bc1_04 | 0.45070422535211274 |
| | None |
| Bc2_01 | 0.5076923076923077 |
| Bc2_02 | 0.4927536231884059 |
| Bc2_03 | 0.4626865671641791 |
| Bc2_04 | 0.4647887323943662 |
| Bc2_05 | 0.45070422535211274 |
| Bc2_06 | 0.38028169014084506 |
| | None |
| | None |
| | None |
| | None |
| | None |
| | None |
| | None |
| | None |
| Bc4_01 | 0.38235294117647056 |
| Bc4_02 | 0.6197183098591549 |
| Bc4_03 | 0.5915492957746479 |
| Bc4_04 | 0.5857142857142857 |
| Bc4_05 | 0.5245901639344263 |
| Bc4_06 | 0.5797101449275363 |
| | None |
| | None |
| Bc5_01 | 0.39436619718309857 |
| Bc5_02 | 0.45714285714285713 |
| Bc5_03 | 0.4788732394366197 |
| Bc5_04 | 0.4788732394366197 |
| Bc5_05 | 0.4852941176470588 |
| Bc5_06 | 0.4782608695652174 |
| Bc5_07 | 0.5492957746478874 |
| | None |
| Bc6_01 | 0.5357142857142857 |
| Bc6_02 | 0.5362318840579711 |
| Bc6_03 | 0.5070422535211268 |
| Bc6_04 | 0.4647887323943662 |
| Bc6_05 | 0.4666666666666666 |
| Bc6_06 | 0.43661971830985913 |
| Bc6_07 | 0.5797101449275363 |
| Bc6_08 | 0.5142857142857142 |
| | None |
| | None |
| | None |
| | None |
| | None |
| | None |
| | None |
| Bc8_01 | 0.4516129032258064 |
| Bc8_02 | 0.49295774647887325 |
| Bc8_03 | 0.5142857142857142 |
| Bc8_04 | 0.5 |
| Bc8_05 | 0.5245901639344263 |
| Bc8_06 | 0.5625 |Figure S2: Transmission frequencies of B. juncea and B. carinata B-genome SSR marker alleles from a near-allohexaploid hybrid to a population of microspore-derived lines. Horizontal dotted lines show thresholds for significant deviation from 50% inheritance (p<0.05, Pearson’s chi-squared test). * Chromosomes Bc3 and Bc7 were absent in the near-allohexaploid hybrid.

## Slide 3
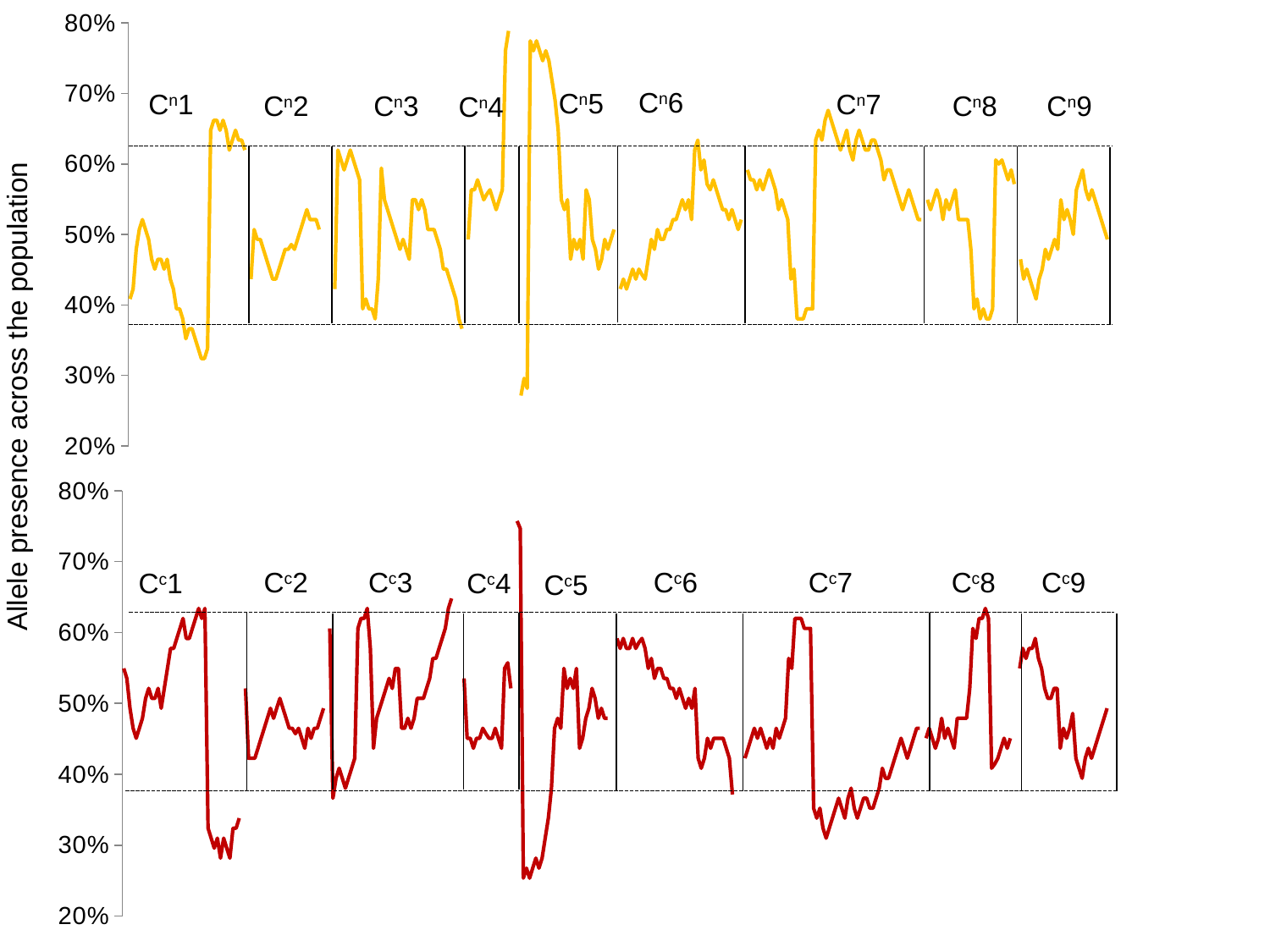

### Chart
| Category | |
|---|---|Cn6
Cn5
Cn1
Cn7
Cn9
Cn2
Cn3
Cn8
Cn4
Allele presence across the population
### Chart
| Category | |
|---|---|Cc3
Cc6
Cc2
Cc7
Cc8
Cc9
Cc1
Cc4
Cc5
Figure S3: Transmission frequencies of B. juncea and B. carinata B-genome SSR marker alleles from a near-allohexaploid hybrid to a population of microspore-derived lines. Horizontal dotted lines indicate significant deviation from 50% inheritance (p<0.05, Pearson’s chi-squared test).

## Slide 4
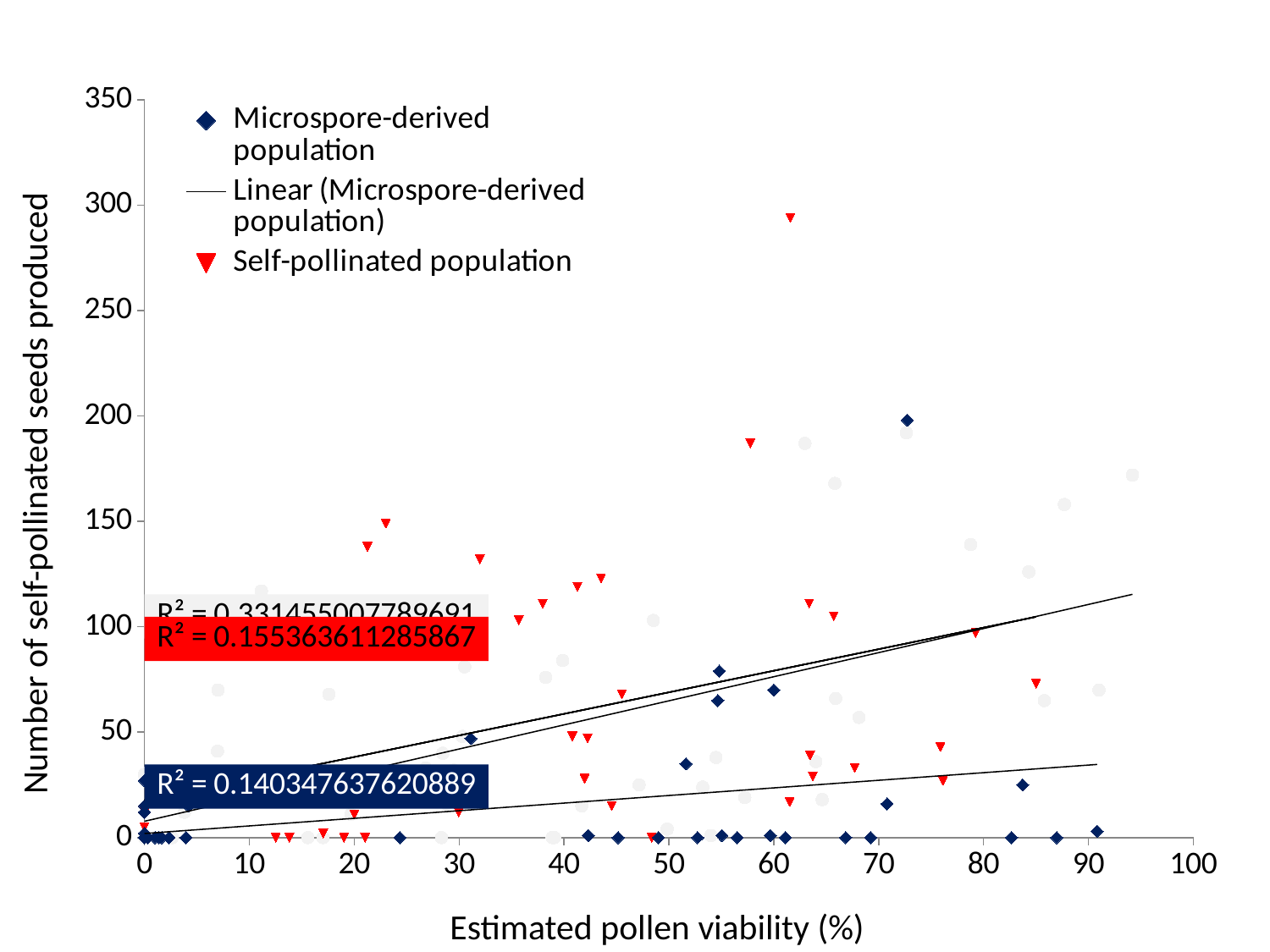

### Chart
| Category | | | |
|---|---|---|---|Number of self-pollinated seeds produced
Estimated pollen viability (%)
Figure S4: The relationship between pollen viability estimates and self-pollinated seed production in three populations derived from microspore culture, self-pollination and open-pollination of the same trigenomic hexaploid hybrid resulting from the cross (B. napus x B. carinata) x B. juncea.

## Slide 5
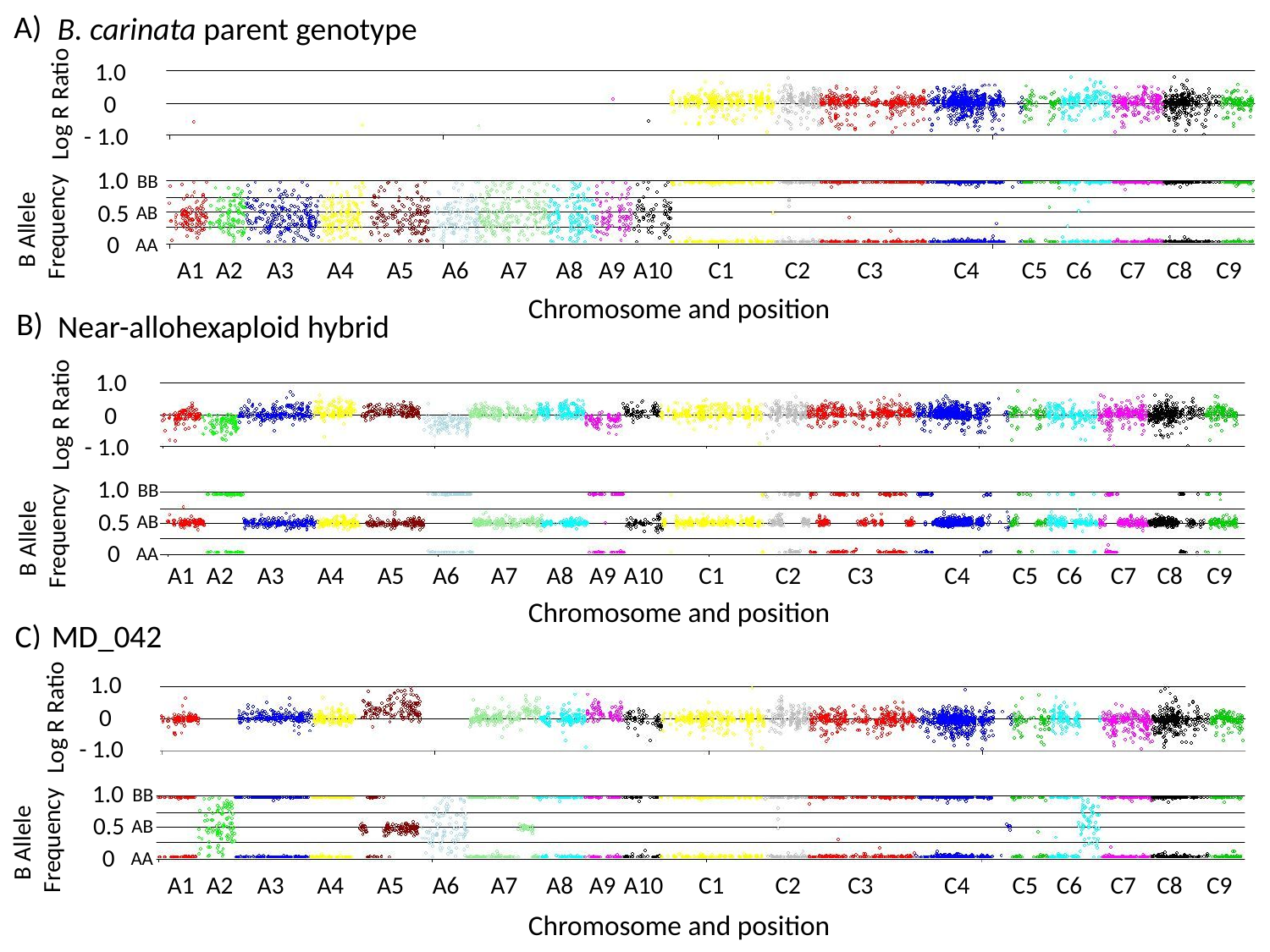

A)
B. carinata parent genotype
1.0
0
Log R Ratio
- 1.0
1.0
BB
B Allele
Frequency
0.5
AB
0
AA
A1
A2
A3
A4
A5
A6
A7
A8
A9
A10
C1
C2
C3
C4
C5
C6
C7
C8
C9
Chromosome and position
B)
Near-allohexaploid hybrid
1.0
0
Log R Ratio
- 1.0
1.0
BB
B Allele
Frequency
0.5
AB
0
AA
A1
A2
A3
A4
A5
A6
A7
A8
A9
A10
C1
C2
C3
C4
C5
C6
C7
C8
C9
Chromosome and position
MD_042
C)
1.0
0
Log R Ratio
- 1.0
1.0
BB
B Allele
Frequency
0.5
AB
0
AA
A1
A2
A3
A4
A5
A6
A7
A8
A9
A10
C1
C2
C3
C4
C5
C6
C7
C8
C9
Chromosome and position
Figure S5: Copy number variation for A and C genome SNP alleles. Log R Ratio is averaged for all SNPs within each individual such that dots (SNPs) above the line indicate higher copy number for those SNPs relative to other SNPs and dots below the line indicate lower copy number of those SNPs relative to other SNPs. B Allele Frequency refers to the ratio of A:B allele amplification, where a ratio of 0.5 indicates a heterozygote (AB) and 0 or 1 indicates homozygosity (presence or absence of the B allele). A) Absence of all A genome chromosomes and presence of two identical copies of each C genome chromosome in a B. carinata line. B) One copy of chromosomes A2, A6 and A9 and two copies of all other chromosomes in a near-allohexaploid hybrid. C) Example microspore-derived line MD_042 from the near-allohexaploid hybrid, with absence of chromosomes A2, A6 and part of C6, an extra copy of chromosome A5 and a duplication of part of chromosome A7.
